# Complex foraging behaviours in wild birds emerge from social learning and recombination of components

**DOI:** 10.1098/rstb.2020.0307

**Published:** 2022-01-31

**Authors:** S. Wild, M. Chimento, K. McMahon, D. R. Farine, B. C. Sheldon, L. M. Aplin

**Affiliations:** ^1^ Cognitive and Cultural Ecology Research Group, Max Planck Institute of Animal Behavior, Am Obstberg 1, 78315, Radolfzell, Germany; ^2^ Centre for the Advanced Study of Collective Behaviour, University of Konstanz, Konstanz, Germany; ^3^ Edward Grey Institute, Department of Zoology, University of Oxford, South Parks Road, OX1 3SZ Oxford, UK; ^4^ Department of Evolutionary Biology and Environmental Science, University of Zurich, Zurich, Switzerland; ^5^ Department of Collective Behavior, Max Planck Institute of Animal Behavior, Universitätstrasse 10, 78464 Konstanz, Germany

**Keywords:** cumulative cultural evolution, social learning, social networks, *Parus major*, animal culture, NBDA

## Abstract

Recent well-documented cases of cultural evolution towards increasing efficiency in non-human animals have led some authors to propose that other animals are also capable of cumulative cultural evolution, where traits become more refined and/or complex over time. Yet few comparative examples exist of traits increasing in complexity, and experimental tests remain scarce. In a previous study, we introduced a foraging innovation into replicate subpopulations of great tits, the ‘sliding-door puzzle’. Here, we track diffusion of a second ‘dial puzzle’, before introducing a two-step puzzle that combines both actions. We mapped social networks across two generations to ask if individuals could: (1) recombine socially-learned traits and (2) socially transmit a two-step trait. Our results show birds could recombine skills into more complex foraging behaviours, and naïve birds across both generations could learn the two-step trait. However, closer interrogation revealed that acquisition was not achieved entirely through social learning—rather, birds socially learned components before reconstructing full solutions asocially. As a consequence, singular cultural traditions failed to emerge, although subpopulations of birds shared preferences for a subset of behavioural variants. Our results show that while tits can socially learn complex foraging behaviours, these may need to be scaffolded by rewarding each component.

This article is part of a discussion meeting issue ‘The emergence of collective knowledge and cumulative culture in animals, humans and machines’.

## 1. Introduction

Once thought to be almost exclusively human, research has now extended the taxonomic reach of culture [1], with cultural traits identified across a range of species and behavioural domains [2], from sleeping patterns in meerkats [3] to territorial song in passerines [4]. Similarly to humans, these animal cultures are not static, but can change over time in response to Darwinian-like processes such as drift and cultural selection [5,6]. Furthermore, in addition to change in diversity or distribution, recent evidence suggests that animal cultural traits themselves can potentially be shaped by cultural evolution, becoming more refined across generations of learners via a process of repeated innovation, modification and social learning [7]. Outside of bird song [8–11], perhaps the most compelling evidence for this stems from migration routes in ungulates [12] and cranes [13]. In both cases, reintroduced populations had largely lost their migration routes, but gradually regained migratory routes that then became straighter and better matched to the environment over generations. This was likely via a combination of individual gains in experience that were then transmitted to younger generations [13,14] and an ongoing collective pooling of knowledge across individuals [15,16].

The outstanding characteristic of human culture is its ability to become progressively refined and/or complex over time via a process of cumulative cultural evolution (CCE) [17], allowing populations to develop increasingly complex skills and technology [18]. Mesoudi & Thornton [19] recently formalised a two-part definition for describing CCE. First, a *core criterion* describes changes in behaviour to improve performance and then social transmission of this behaviour, with these steps repeated for sequential improvements over time. A so-called *extended criterion* describes more ‘complex’ or ‘advanced’ forms of CCE, including having multiple functionally dependent traits, diversification of cultural lineages, or recombination across lineages. While still highly controversial, evidence from non-human animals (including those examples above) now suggests that the core criteria of CCE might be widespread [5,8,12,15,20–22]. Yet, it is also clear that there is little or no evidence for the extended criteria in non-human animals, outside of a few possible examples in bird and cetacean song [5,8,23]. Why this is the case is hotly debated [1,24–26].

There is extensive debate about what social, cognitive or demographic factors underpin CCE in humans, but it is becoming increasingly clear that the ability to recombine traits across cultural lineages into more rewarding behaviours is a fundamental component [19,27–29]. To persist over generations, these composite or functionally dependent traits then have to be transmitted onwards via social learning. The evolution of complex culture has therefore long been argued to also depend on high fidelity copying mechanisms that will help retain modifications, such as imitation and teaching [30–33], and there is now an extensive body of experimental work testing the role of these mechanisms for CCE in humans [34–36]. Comparative studies with humans and other primates have also shown that learning mechanisms (e.g. imitation and teaching) differ across primates with CCE and those without, giving further indirect evidence for the importance of transmission fidelity [30,31,37–39]. Yet there is also recent evidence that alternative mechanisms can underlie the emergence of CCE-like patterns. For example, in a transmission chain experiment in which Guinea baboons and children performed a non-copying task, Saldana and colleagues found that task performance and systematic structure increased over generations of learners of both species, questioning the role for transmission fidelity in CCE [40,41].

In perhaps, the most comprehensive experiment examining the mechanisms underlying CCE in non-human animals, Dean *et al*. [42] presented a three-step puzzle-box that gave increasing rewards for solving progressive steps to groups of human children, chimpanzees and capuchins. Only human children were able to consistently reach step 2 or 3, which was attributed to presence of teaching and prosocial behaviours. It should be noted that a follow-up study demonstrated that children were capable of solving this task with asocial learning alone, although it remains likely that teaching, imitation and prosociality facilitated success [43]. More recently, Vale *et al*. [44] examined the likelihood of transmission after a recombination event, by introducing demonstrators trained on a tool modification into groups knowledgeable about a simpler task. Chimpanzees were able to learn increasingly complex behaviours via social learning, suggesting that the ability to do so is present, at least in our nearest relatives. However, there is a paucity of experimental work examining the full pathway from innovation to recombination to transmission in non-human species. Such experiments are vital to identify the ‘weak links’ that prevent most non-human species from exhibiting complex cultures.

Great tits (*Parus major*) are well known to be highly innovative and opportunistic [45–48]. A long history of research has also shown that great tits rely on social learning across multiple behavioural domains, from song [49] to breeding decisions [50], to where and what to eat [51–53]. In a previous study, we performed a cultural diffusion experiment in replicate subpopulations of great tits where we introduced a novel foraging behaviour of sliding a door to left or right to solve a foraging puzzle [54]. Once introduced, knowledge of how to access this resource using this foraging technique spread rapidly via social learning [54]. Side preferences were sustained over generations as cultural traditions [54,55]. Here, we diffused a new tradition for turning a dial to solve a foraging puzzle into the same subpopulations, before giving individuals the opportunity to recombine these traits into a more complex two-step puzzle-box to gain progressively higher rewards. We then reinstalled two-step puzzle-boxes in the following generation to test whether the naïve juveniles could learn the more complex task without experience of rewarding singular components. This three-stage experimental design allowed us to examine the full pathway from recombination to over-generational transmission by asking (1) whether tits can recombine socially learned behaviours into more rewarding traits; (2) whether they can socially transmit more complex traits within and between generations; and if so, (3) how this affects emergence of local traditions.

## 2. Methods

### (a) Study system

Our experiment was conducted over two winters, from November 2015 to February 2017, in a population of great tits (*Parus major*) at the Wytham Woods study site near Oxford, UK (*ca* 51°84′° N, 01° 82′° W) The majority of a population of approximately 1000 great tits in Wytham Woods are fitted with both a British Trust for Ornithology (BTO) unique metal leg-ring and a plastic leg-ring fitted with a passive-integrated transponder (PIT) tag with a unique identifier code (IB-Technology). All birds are additionally sexed and aged based on plumage and breeding records [56]. In winter, great tits form loose fission–fusion flocks that exploit ephemeral food sources such as beech mast [56]. During this time, birds will also readily exploit provisioned food at bird feeders. Thus, radio-frequency identification (RFID) antennas built into the perches at these feeder stations can be used to detect PIT-tagged birds and record individual visits [57].

**Figure 1.**
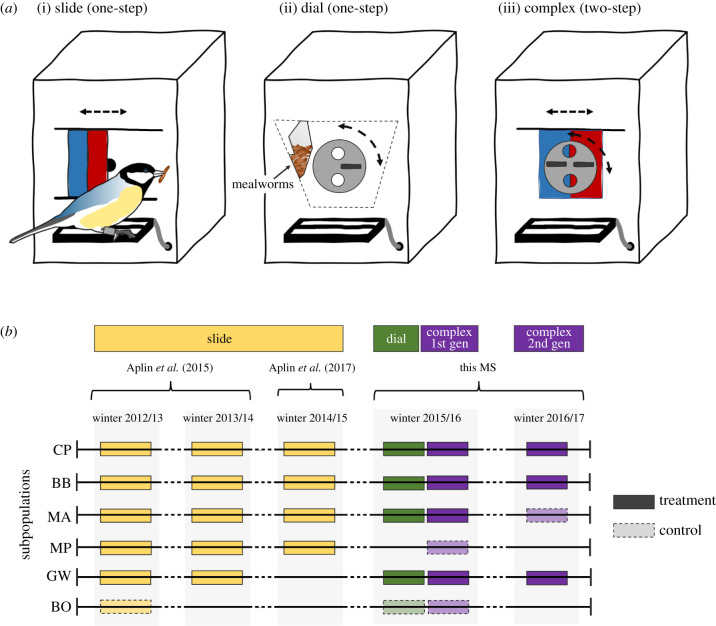
Puzzle box design and experimental timeline. (*a*) The three foraging tasks used in this study. All had an internal printed circuit board, RFID antenna and data logger, such that the identity and action of visiting birds were recorded, and the door reset after each visit. (i) Two-action *sliding-door puzzle-box*, where birds could push the door left or right to expose a mealworm feeder (sliding door experiment). (ii) Two-action *dial puzzle-box*, where birds could spin a circle counter-clockwise (left) or clockwise (right) to expose a mealworm feeder (dial diffusion experiment). (iii) Twelve-action *progressive two-step puzzle-box*, where birds could perform one one-step solution (slide left/right, or dial counter-clockwise/clockwise) to receive a sunflower seed or combine one variant of dial and slide together in any order to produce a two-step solution (e.g. dial left + slide right), rewarded by a mealworm (complex first generation experiment). This puzzle-box was then modified into a *full two-step puzzle-box* by providing no reward for single actions (used in complex second generation experiment). (*b*) Schematic timeline of slide, dial and two-step diffusion experiments across six replicate subpopulations, incorporating previously published work [54,58]. (Online version in colour.)

In a series of previous studies running from November 2012 to March 2015, we introduced a novel foraging resource—the *sliding-door puzzle-box* (figure 1*a*i)—where birds could move a small door either to the left or right to expose a mealworm feeder (a highly preferred food type). We first performed a cultural diffusion experiment [59], installing three puzzle-boxes 250 m apart into the eight subpopulations, and then introducing birds trained on one of the two alternative solution variants (slide-left or slide-right) into five treatment subpopulations (figure 1*b*). The final three subpopulations acted as controls, with no knowledgeable birds introduced. In total, 414 individuals learned the behaviour [54], with populations strongly biased to the introduced variant. Second, puzzle-boxes were reinstalled in the five treatment subpopulations in the following year (figure 1*b*), with behaviour rapidly transmitting to the second generation [54]. Finally, four of these subpopulations underwent a further experiment in November 2014–March 2015 (figure 1*b*), where the rewards for the two solving actions were varied [58], mimicking a changing resource environment. This did not change knowledge of how to access the puzzle-boxes, but led to approximately 50% of individuals changing their preference for left or right. Annual survival of great tits is approximately 50%. Therefore, given these previous studies, we expected that 20–40% of the general population would still know how to solve the sliding-door puzzle-box in winter 2015–2016.

### (b) Experimental apparatus

The experimental apparatus consisted of three different puzzle-boxes, all based on the puzzle-box design used in Aplin *et al.* [54] and all automated with the help of a printed circuit board (Stickman Technology, UK), movement sensors, motor, and RFID-antenna (figure 1*a*). In the first dial puzzle-box, access to the bird feeder was blocked by a circular dial that could be rotated counter-clockwise (left) or clockwise (right) to line up an embedded hole with access to live mealworms (a highly preferred food) (figure 1*a*ii). In the second progressive two-step puzzle-box, this dial was combined with the previous sliding door to create a two-step task (figure 1*a*iii). Birds could complete one action (i.e. slide left/right, or dial left/right) to reveal a feeding hole containing sunflower seed (a moderately preferred food), while if they added a second action they could reveal a second feeding hole containing the more preferred food of live mealworms. This gave a total of 12 possible rewarding solutions (four variants of low-rewarding one-step solutions: 1: dial-left, 2: dial-right, 3: slide-left, 4: slide-right; and eight high-rewarding solutions: 5: dial-left + slide-left, 6: dial-left + slide-right, 7: dial-right + slide-left, 8: dial-right + slide-right, 9: slide-left + dial-left, 10: slide-left + dial-right, 11: slide-right + dial-left, 12: slide-right + dial-right). In a third full two-step puzzle-box (figure 1*a*iii), the reward for performing one-step solutions was removed, but birds were rewarded for producing any of the eight variants of the two-step solution.

### (c) Experimental design

#### (i) Dial diffusion experiment

Between 1 December 2015 and 4 January 2016, we caught two male great tits at four subpopulations in Wytham Woods (figure 1*b*), and trained them in captivity to solve the dial puzzle-box by turning the circle counter-clockwise (‘dial left’; subpopulations CP and MA) or clockwise (‘dial right’; BB and GW) (figure 1*a*). These birds were released back into their subpopulations to act as initial demonstrators, and three baited puzzle-boxes were installed 250 m apart in each area (apart from GW, where only one puzzle-box was installed). In a fifth subpopulation (BO), three puzzle-boxes were installed, but no birds were trained (figure 1*b*)—as in Aplin *et al*. [54], this population acted as a control to examine the rate of asocial learning. Puzzle-boxes were kept continuously baited for 20 days over four weeks (Monday to Friday), and removed on weekends to allow for measurement of social networks, cleaning, data download, and for puzzle-boxes to be rotated between sites (controlling for any potential differences between boxes).

#### (ii) Complex first generation experiment

Immediately following this first stage of the study, a progressive two-step puzzle-box was installed for 15 days over three weeks (figure 1*b*). This task combined the elements of both the sliding-door puzzle and the dial puzzle, with 12 possible solution variants. Four of these variants (one-step solutions) gave a moderate reward of sunflower seed, while solving both dial and slide together (two-step solutions) exposed the more preferred mealworm reward. No additional birds were trained on how to solve this task. Instead, populations contained a mix of individuals that were either (i) knowledgeable about both components (dial and slide), (ii) knowledgeable about dial or slide only or (iii) completely naïve. As a few birds had learned to dial in the control subpopulation BO, this was used as a partial control to examine the acquisition of the two-step solution in areas with no existing knowledge of one component (slide). In addition, we added a further partial control subpopulation in MP, an area with existing knowledge of slide, but no knowledge of dial (figure 1*b*).

#### (iii) Complex second generation experiment

Lastly, puzzle-boxes were reinstalled at the four treatment subpopulations in the following winter (2016/17) for 20 days over four weeks (figure 1*b*). For this final stage of the experiment, no reward was given for one-step solutions, so birds had to perform the entire two-steps of the full two-step puzzle-box to be rewarded. No additional birds were trained on solving the puzzle, but we expected the subpopulations to consist of (i) naïve juveniles hatched in the intervening spring, (ii) older individuals with no, or partial, knowledge and (iii) older individuals with full knowledge of the two-step behaviour. In one treatment site, MA, none of the knowledgeable individuals from the previous winter were observed again in this period, and so it was treated as a control subpopulation.

### (d) Social networks

During each experimental period, eight openly accessible bird-feeding stations were deployed around each subpopulation in a 250 × 250 m grid. These stations opened from dawn to dusk Saturday and Sunday for the weekends within and surrounding each period, collecting data for ten (dial diffusion and complex second generation experiments) or 8 days (complex first generation experiment). Each contained sunflower seeds and was equipped with two feeding holes surrounded by RFID antennas and a data logger to record all visits by PIT-tagged individuals. To detect social groupings in the spatio-temporal patterning of these visits, we used a Gaussian mixture model to identify clusters in the data stream that are likely to represent social flocks [57,60]. Associations were then calculated with a ‘gambit of the group’ approach, where birds were associated if observed in the same flock, and association strength calculated using the simple ratio index [61–63]. Finally, to deal with biases arising out of variation in observability, we restricted analyses to birds with a minimum of ten observations at the feeding stations. Social networks calculated in this way have been demonstrated to be consistent over time [54] and have functional significance for the individuals embedded in them, including for the transmission of information [53,64,65] and behaviour [54]. All network analyses were conducted in R, using the packages asnipe [66], sna [67], and igraph [68].

### (e) Network-based diffusion analysis

To investigate the importance of social learning for acquisition of the different solution categories (dial, slide and two-step), we ran network-based diffusion analyses (NBDAs) on the three stages of the experiment [69–71]. For all analyses, we ran the ‘time of acquisition diffusion’ (TADA) variant of NBDA. This considers the times at which individuals first acquired a behaviour as diffusion data [70,71], and compares this diffusion data against the social network to identify whether social transmission of the behaviour is likely to have occurred.

We conducted nine different NBDAs (electronic supplementary material, table S1) to investigate the importance of social learning on the diffusion of the different solution categories across the three experiments and investigate pathways of transmission. In the dial diffusion experiment, we used the time at which individuals acquired a dial variant as diffusion data. In the complex first generation experiment, we investigated diffusions of dial, slide and two-step solutions in three separate NBDAs. Similarly, we ran three NBDAs for the diffusions of dial and slide components, as well as two-step solutions for the complex second generation experiment. In the complex second generation experiment, we were additionally interested in the pathways of transmission, asking whether birds were able to learn dial or slide components from observing two-step solves, or whether they learnt these from other partial solves even though one-step solutions were not rewarded in this experiment. To differentiate between the two pathways, we ran NBDA with two networks. In the first network, naïve individuals could only learn one-step solutions from those producing two-step solutions, while in the second network, birds could only learn the one-step solutions from those also producing the respective one-step solution. We integrated this into a dynamic network framework, in which the network was updated at each acquisition event depending on the knowledge status of each bird (whether it produced one-step or two-step solutions).

We considered individuals as knowledgeable if they had produced at least three solutions within a category at least three times during a diffusion experiment. Birds with prior knowledge in solving the task at the start of each experiment were included as demonstrators in the models (SI). For the dial diffusion experiment, this was one bird per site, trained in captivity. For the complex first generation experiment (dial and slide diffusions), these were birds that had produced dial or slide in previous experiments (including [54,58]), whereas for the diffusion of the two-step solution, we did not include any demonstrators as none of the birds had been trained on the full task combination. Birds moving between subpopulations and solving the puzzle in more than one (overlaps between CP, BB, GW; *N* = 19 dial, *N* = 5 slide, *N* = 3 two-step) were only considered as learners in the subpopulation in which they first showed the behaviour. Finally, for the complex second generation experiment, for all diffusions (dial, slide, two-step), we included all individuals as demonstrators that had successfully solved the respective task at least three times in previous experiments.

NBDA allows the inclusion of individual-level variables (ILVs) that could potentially influence the social and asocial learning rate [70,71]. For diffusions of dial and slide solutions (in the dial diffusion, complex first and second generation experiment), we included sex (−0.5 for females; 0.5 for males; 0 for unknown) and age (−0.5 for first year; 0.5 for adults) (electronic supplementary material, table S1). For the diffusion of the two-step task (first and second generation experiments), we additionally included two time-varying variables depicting the birds' experience level at each time step throughout the experiment as experience with either dial or slide, or with both components but not in combination. This allowed us to control for a situation in which birds may rely on social learning to acquire the single components, but may then recombine components asocially. Because few birds learned the two-step solution in the complex second generation experiment, we dropped sex and age as ILVs to improve model fitting. In all models, we allowed ILVs to influence asocial and social learning rate independently [70,72].

We fitted models using NBDA version 0.9.6 [73] in R v. 4.0.5 [74], with and without social learning, and with all possible combinations of the respective ILVs (electronic supplementary material, table S1). For each task, we combined all treatment subpopulations in a TADA with multiple diffusions (electronic supplementary material, table S1). Note that for the complex first generation experiment, we included the control site (MP), as some birds, which were prior dial learners in MA, started producing the two-step solution after an innovation event and we found that including the subpopulation improved model fitting. For the complex second generation experiment, only BB had enough new learners for the number of parameters we aimed to include, and so TADA was only performed in this subpopulation. For each task, we estimated the strength of the social learning effect conditional on the best performing social model by AICc (Akaike information criterion corrected for sample size) [75], by extracting estimates for the social learning parameter *s.* This parameter is defined as the strength of social transmission per unit association with informed individuals relative to the baseline rate of asocial learning [70,71]. We set the baseline rate of asocial learning to an individual at the midpoint of all ILVs. We further extracted the effects of each supported ILV (those with summed AICc weights $\left(\sum w_{i}\right)$ of >0.5) on the social and asocial learning rate as model averaged estimates (medians), and derived 95% confidence intervals for each parameter using profile likelihood techniques [76] based on the best predictive model.

### (f) Other statistical analysis

As a supporting analysis that also included birds not present in the social network, we used a logistic regression to test whether the knowledge state of individuals within the complex first generation and complex second generation experiments predicted whether they would learn any two-step solutions. Control populations were excluded, and birds were considered to have learned any category of solution (dial, slide, two-step) if they had produced three or more solutions of that category within an experiment. Birds that learned the two-step solution in the complex first generation experiment, were not counted again as data points in the complex second generation experiment. We performed a logistic general linear mixed model (LMM) [77] in which learning the two-step solution was predicted by a full interaction between binary variables of prior experience with dial and prior experience with slide.

To measure behavioural diversity in birds’ solution variants choice, we calculated Shannon entropy using the set of solution variants within individual birds, within sites and within subpopulations [78]. Shannon entropy, often used as a measure of biodiversity, is a measure of the amount of information needed to describe a set of types of varying frequency, with larger entropy values indicating a less structured, more randomly distributed set. Entropy was then exponentiated to produce an individual behavioural uncertainty score. In the case of a uniform distribution of variants within a set, or the maximum entropy distribution, exp(H) is equal to the number of types present in the set. In the case of a non-uniform distribution, exp(H) approaches the number of dominant types in the set. For example, if a bird has produced *n* variants, a behavioural uncertainty score near 1 would indicate that most of the bird's behavioural productions were of one type. As above, a bird was only included if it produced three or more solutions within a solution category.

To compare the observed behavioural diversity within sites and subpopulations against those expected under similar conditions with no social learning, we permuted the data 10 000 times, swapping solution variants within individuals, but keeping their distribution of production the same. To test how likely the distribution of observed behavioural diversity was within individuals (i.e. compared to if they had no variant preferences), we randomized every production within an individual, choosing with replacement from the set of their known two-step solving techniques, and repeated this 10 000 times to create a dataset of simulated individuals. In both types of permutation tests, we reported tail probabilities to assess how likely the observed values were within the distribution of values generated through permutations. Tail probability represents the probability that the simulation would produce a value below the observed value, derived from the empirical cumulative distribution function (ECDF).

## 3. Results

### (a) Summary

**Figure 2.**
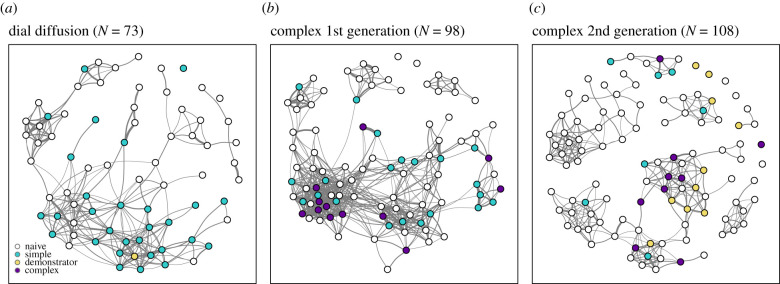
Social association networks at subpopulation BB (*a*) for the dial diffusion, (*b*) for the complex first generation experiment and (*c*) for the complex second generation experiment. Nodes represent individual birds and are coloured according to state of knowledge. Links between nodes represent the strength of the connection between two individuals. Yellow nodes are knowledgeable demonstrators (either trained in captivity or solving in the previous generation), white nodes are naïve individuals. All other nodes are coloured according to their final knowledge state gained, with blue = dial/slide, and purple = complex solvers. (Online version in colour.)

**Figure 3.**
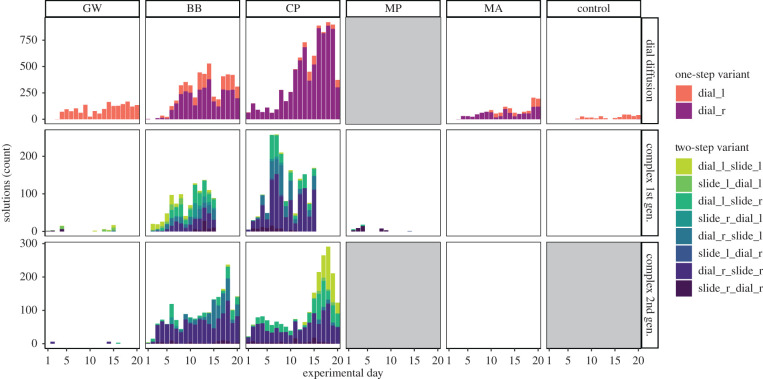
Overview of diffusion and production of solutions across subpopulations and experiments. Solution variants are coloured, and while one-step solutions (dial and slide) continued to be produced in the complex first and second generation experiments, for ease of viewing, only two-step solutions are shown in these stages. GW and BB were initially seeded with ‘dial L’ (counter-clockwise), while CP and MA were seeded with ‘dial R’ (clockwise). The height of bars represent the number of total solutions produced per day by all birds. Experimental day is shown on the *x*-axis, ranging from 1–20 in dial and complex second generation experiments, and 1–15 in the complex first generation experiment. NB: As there were no knowledgeable birds present to act as demonstrators in MA in the complex second generation experiment, it was used as the control site. (Online version in colour.)

In treatment subpopulations for the dial diffusion experiment, an average of 21.9% of birds that had been recorded on the puzzle-box subsequently solved the task (table 1(i) and figure 2). Across the four treatment subpopulations, birds produced a total of 18 426 dial solutions (table 1(ii)) with a significant bias towards the variant introduced by the demonstrator at three (CP, GW, MA) out of the four sites (Welch *t*-test; *t* = −5.93; *p* = 0.001; table 1(iii) and figure 3). The fourth subpopulation (BB), showed a bias towards the non-seeded variant (table 1(iii) and figure 3). This likely occurred because of an independent innovation event of the alternative variant at a different puzzle-box on the same day as the demonstrator began to solve. This variant spread faster and eventually dominated.

**Table 1.** Summary table including numbers of solves and percentages of birds solving at the subpopulation level across the three experimental stages.

	**BB**	**CP**	**GW**	**MA**	**MP**	**BO**
*dial diffusion* (*single-step task*)						
(i) % birds solving the one-step task	34.7	29.2	12.9	10.7	—	9.5
(ii) no. solves of the one-step task	5340	8522	2646	1607	—	306
(iii) bias towards demonstrator solution (side) in the one-step task	0.34	0.94	0.99	0.75	—	NA
*complex first generation experiment* (*progressively rewarded two-step task*)						
(iv) % birds solving the one-step component(s)	16.7	22.1	16.7	13.6	10.5	11.8
(v) no. solves of one-step components	4546	7400	1706	1680	1804	352
(vi) % birds solving the full two-step task	8.9	10.3	2.9	2.1	6.7	0
(vii) no. solves of the full two-step task	1219	1815	182	58	51	1
*complex second generation experiment* (*two-step task with only full solves rewarded*)						
(viii) no. birds present in second year that were two-step solvers in the first generation	13	3	1	0	—	—
(ix) % naïve birds solving the (unrewarded) one-step component(s)	4.9	9.8	7.6	9.8	—	—
(x) no. solves of one-step components	2148	2105	482	164	—	—
(xii) % naïve birds solving the full two-step task	18.0	7.8	4.5	0.0	—	—
(xiii) no. solves of the two-step task	2164	2124	484	0	—	—

In the complex first generation experiment where the one-step and two-step solutions gave progressively higher rewards, an average of 17.2% of birds that visited a puzzle-box only learned one or both of the one-step solutions, producing a total of 17 488 solves (12 319 dial, 5169 slide) (table 1(iv–v); figures 2 and 3). A further average of 5.7% of birds learned the two-step solution, totalling 3316 solves (table 1(vi–vii); figures 2 and 3). At one control subpopulation, by contrast, no birds produced two-step solutions (table 1(vi) and figure 3).

Finally, in the complex second generation experiment where only two-step solutions were rewarded, 17 birds were present that had performed two-step solutions in the previous winter, and continued to produce them (table 1(viii)). An additional 14 naïve birds learned the full two-step solution in the second generation experiment. Altogether, birds produced 4772 two-step solutions (table 1(xii) and figure 3). Interestingly, birds also continued to perform the unrewarded single components, with 4899 single components produced across the three subpopulations (3775 dial, 1124 slide; table 1(ix–x); figures 2 and 3). No birds learned the two-step solution at the control subpopulation (MA) (table 1(xii) and figure 3).

### (b) Network-based diffusion analysis

There was strong support for social ($\sum w_{i}>0.99$) over asocial models in the analysis of the dial diffusion experiment. In the best model, 85.8% [95% CI: 77.2–92.8%] of birds learned the dial task through social learning. The social learning rate was influenced by both age and sex (electronic supplementary material, table S1), with adults being 2.0 [95% CI: 1.0–4.4] times faster at learning compared to juveniles, and males being 2.5 [95% CI: 1.3–5.3] times faster than females.

In the complex first generation experiment, 18.6% and 30.7% of the general population present were knowledgeable in solving slide and dial, respectively, prior to the start of the experiment. For new learners of the one-step solutions, we also found strong support for social transmission of both the dial ($\sum w_{i}>0.99$) and slide solutions ($\sum w_{i}>0.99$), with adults being faster at learning both one-step tasks compared to juveniles, and males being faster at learning compared to females (electronic supplementary material, table S1). Overall, 91.5% of all learners were estimated to have learned dial through social learning [84.6–100%], while 71.0% were estimated to have learned slide socially [56.2–100%]. Meanwhile, for the acquisition of the full two-step task, we found most support for asocial ($\sum w_{i}=0.53$; *N* = 16) over social models ($\sum w_{i}=0.47$; *N* = 256; electronic supplementary material, table S1). Males were an estimated 3.2 [95% CI: 1.5–7.1] times faster at acquiring the complex task asocially compared to females, and birds with prior knowledge of one component were 24.0 [95% CI: 9.3–81.8] times faster at learning the two-step task asocially compared to those without any knowledge, while those with knowledge of both components were 35.6 [95% CI: 12.7–127.4] times faster at learning asocially compared to those with no knowledge or knowledge of only one (electronic supplementary material, table S1). This suggests that (i) birds were recombining their previous knowledge of the individual components to acquire the full two-step behaviour, with no additional social input required to make this step; and (ii) birds that had previous knowledge of one step of the task were still able to asocially reconstruct the full task, albeit with a longer latency to do so. Further evidence that birds were using previously socially learned knowledge in this process comes from solution choice. Birds tended to use the same dial variant in this experiment as they acquired in the first dial diffusion experiment, and within this experiment, birds tended to use the same technique variants when performing one step or two steps.

Finally, in the complex second generation experiment, we again found most support for social transmission of both dial ($\sum w_{i}=0.92$) and slide ($\sum w_{i}=0.90$; electronic supplementary material, table S1). Overall, 100% of birds that learned to solve dial [64.6–100%] and 78.0% of those that learned to solve slide [36.9–88.3%] were estimated to have done so through social learning. For dial, the multi-network NBDA revealed most support for a social transmission pathway from other birds solving dial $\left(\sum w_{i}=0.64\right)$, followed by models with transmission from birds solving the two-step task ($\sum w_{i}=0.17$; electronic supplementary material, table S1). For slide, we found most support for a social transmission pathway from birds solving the two-step task ($\sum w_{i}=0.55$), followed by models with transmissions from both two-step solvers and those solving slide only ($\sum w_{i}=0.15$; electronic supplementary material, table S1). Age and sex did not influence any of the social or asocial learning rates (electronic supplementary material, table S1). For the diffusion of the two-step task, we again found most support for asocial models ($\sum w_{i}=0.64$; *N* = 4) over social models $(\sum w_{i}=0.36;\;N=16$; electronic supplementary material, table S1). Birds with knowledge of one component were 19.3 [5.1–91.2] times faster at producing the two-step solution than those without any prior knowledge (electronic supplementary material, table S1). These results are consistent with the finding in the complex first generation experiment, with birds acquiring knowledge about the one-step solution socially, and knowledge of one solution being sufficient to then reconstruct the full two-step solution without further social information.

### (c) The effect of prior knowledge on acquisition of the complex behaviour

Without knowledge of either dial or slide, birds were unlikely to learn the two-step solution, with an estimated 4.6% [95% CI: 3.5–6.0%] chance of learning (GLM; intercept – *β* ± s.e. = −3.03 ± 0.28, *z* = −10.66, *p* < 0.001; electronic supplementary material, table S2). Knowledge of only dial raised this probability to 27.8% [95% CI: 16.9–42.2%], while knowledge of only slide raised the probability to 16.7% [95% CI: 7.51–33.0%] (GLM; knowledgeable dial – *β* ± s.e. = 2.07 ± 0.36, *z* = 5.82, *p* < 0.001; knowledgeable slide – *β* ± s.e. = 1.42 ± 0.62, *z* = 2.30, *p* = 0.02). Knowledge of both slide and dial elevated the probability marginally, to 28.9% [95% CI: 5.3–74.8%] (GLM; dial:slide : *β* ± s.e. = −1.36 ± 0.73, *z* = −1.86, *p* = 0.06). From this evidence, it seems that knowledge of either dial or slide was beneficial for learning the two-step solution, but knowledge of both brought only a slight increase in the probability of a bird learning the behaviour. Across the complex first and second generation experiments, 13 birds (22% of learners) were able to acquire the two-step solution without having produced either component (electronic supplementary material, table S3), suggesting that a small percentage of birds were able to learn the two-step solution through observation alone. Furthermore, we found that, of the two-step solvers that had produced one-step solutions prior to their first two-step solution (complex first generation: 35 birds, complex second generation: 10 birds), 79.4% of their two-step solutions contained their most preferred one-step variant as a component. Of the two-step solvers that had produced both dial and slide before learning the two-step solution (complex first generation: 13 birds, complex second generation: 0 birds), 65% of their two-step solutions were a combination of their most preferred dial and slide variants. This is above that expected by chance, and together, these results suggest asocial recombination of already socially learned one-step components, rather than transmission of the entire two-step solution.

### (d) Solution choice within individuals, sites and subpopulations

**Figure 4.**
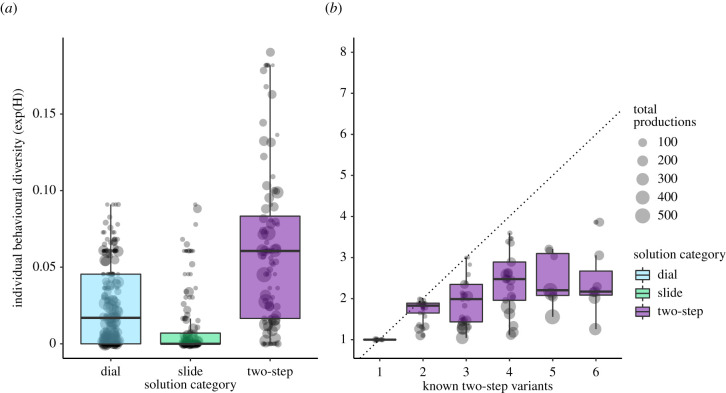
Behavioural diversity in solving techniques. Lower values indicate that an individual preferred fewer solutions, with the number of solutions preferred correlating to the integer value of the score. Each point represents an individual's behavioural productions (size: number of productions), median and quantile values denoted by the box plot. (*a*) Individuals were relatively faithful to their slide and dial variants (dial: median = 1.37; slide: median = 1). Most individuals preferred to produce two out of eight possible two-step variants (median = 1.89). (*b*) Overview of the same measure of individual behavioural uncertainty, compared across how many variants an individual produced over both complex first and second generation experiments. The dotted diagonal line indicates the maximum behavioural diversity score possible if an individual produced all known behaviours with equal frequency. Individuals continued to prefer between two and three two-step variants, even as their number of known variants increased. (Online version in colour.)

There were two equally rewarding alternative variants for each of the one-step solution categories, dial left or right and slide left or right. When considering behavioural productions of one-step solutions (dial and slide), we found that, as expected, individual birds had one preferred variant (figure 4*a*) [54]. Birds were slightly more faithful to their variant choice within slide than dial (LMM; solution category (slide) – *β* ± s.e. = −0.23 ± 0.05, *t* = −4.894, *p* < 0.001; figure 4*a*; electronic supplementary material, table S4). There were eight equally rewarding alternative solving techniques for the two-step task, ranging from dial-left/slide-right to slide-left/dial-right. Birds were significantly less faithful to their technique choice for the two-step task (LMM; solution category (two-step) – *β* ± s.e. = 0.55 ± 0.06, *t* = 10.06, *p* < 0.001), but their estimated diversity score of 1.95 [95% CI: 1.85–2.04] was far below the maximal level (eight), indicating a non-uniform distribution of variant frequencies within birds. The point estimate of an uncertainty score of approximately two can be interpreted as two-step solvers strongly preferring to use two variants out of the eight possible variants. While birds that knew more variants had a significantly increasing diversity score (LMM; no. solutions known – *β* ± s.e. 0.23 ± 0.05, *t* = 4.4.23, *p* < 0.001, electronic supplementary material, table S4), the median value generally remained near two, and far below the maximum possible uncertainty score (figure 4*b*).

**Figure 5.**
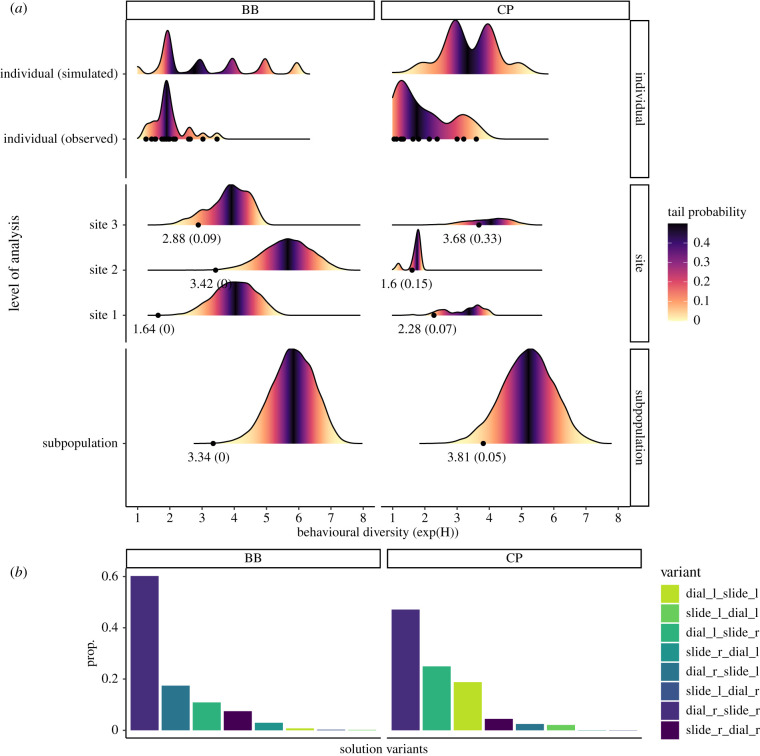
Individual, site and subpopulation level traditions. (*a*) Simulated distributions (fill: tail probability calculated from ECDF) versus observed data points (text: value, tail probability) of behavioural diversity within techniques used for two-step solutions during the complex second generation experiment, at three different levels of analysis. Observed individuals and their simulated data were pooled for easier visualization. Lower tail probabilities, like *p*-values, indicate that observed values are unlikely to be obtained by chance. If there were highly shared preferences at site and population, we would expect a similar level of behavioural uncertainty at individual, site and population levels. We found no evidence for singular traditions. However, observed values generally fell below random expectation at all three levels of analysis, suggesting that (i) individuals preferred to produce only a subset of solutions that they knew and (ii) there were 3–4 shared traditions of higher preference relative to others at the subpopulation level. (*b*) Overview of the proportion of two-step solution variants over time, with dial-right slide-right and dial-left slide-right preferred in both subpopulations, in addition to dial-right slide-left in BB, and dial-left slide-right in CP. (Online version in colour.)

To determine whether there were traditions for any particular two-step variant present at the site or subpopulation levels, we pooled solutions at each of these levels of analysis and calculated a behavioural diversity score, with the expectation that if there were traditions shared among individuals, these scores would remain close to those of individuals. We found no evidence for any singular two-step solution becoming an established tradition (figure 5). However, we found that at the subpopulation level, behavioural diversity fell far below random expectation in both BB and CP (tail probabilities = 0.0, 0.05), indicating that while there was no single dominant solution across a subpopulation, individuals still shared some preference in solutions more than expected by chance. This is consistent with our NBDA results, in that birds were socially learning the components of the two-step solution, but individually learning how to recombine them.

## 4. Discussion

Similar to previous results [54,58], we found that simple foraging behaviours could spread and establish as new foraging traditions in wild great tits, giving further evidence for social learning and the spread of innovation in this species. When provided with the opportunity to chain these traditions together in a foraging task that gave progressive rewards, a proportion of the population were also able to do so. However, birds were generally not able to learn the complex behaviour *ex-nihilo*, and its acquisition instead depended on social learning of the individual components before individual reconstruction of the full complex behaviour. Interestingly, our evidence suggests this continued to be the main learning route in both generations, even though individual components ceased to be rewarded in the second generation and were produced at a lower rate by birds knowledgeable of the full task.

The consequence of the learning pathway to two-step behaviour observed in our study was that, in contrast to simple socially learnt foraging behaviours [21,54], singular population-level traditions failed to emerge. However, sites and subpopulations did share a smaller than expected set of complex behaviours, likely because they were learning socially from a subset of individual components. The combination of social and asocial learning therefore lead to polymorphic cultural traditions at the site and subpopulation level. Overall, our results suggest that complex behaviours can persist in populations, without being necessarily socially transmitted in their entirety. However, judging by the declining transmission rates from the complex first to complex second generation experiment, we expect that the two-step behaviour may not be able to be maintained long-term without the scaffolding provided by the progressive rewarding of each component.

Most birds in our study (nearly 80% of learners, 97% of all birds) did not socially learn the full two-step solution as a complete behaviour. In fact, our results suggest that birds were also unlikely to acquire the complex behaviour from observation of two-step solutions, but instead relied on observing other birds producing the singular components. There may be many contributing factors as to why this was the case. First, cognitive factors, such as limited attention, inhibition or object permanence, might play a role, as learning the complex behaviour required the observation and production of twice as much information. Naturally observed foraging behaviour in tits is often extractive with one action (e.g. pulling bark to reveal insects). However, tits can also spontaneously solve string-pulling tasks, a fairly complex, multi-step process [79]. Second, the low social tolerance and lack of prosocial behaviour in great tits might also play a role by limiting observation opportunities. Prosociality and tolerance has previously been linked with human success at learning a manipulative multi-step task in a previous study comparing cumulative cultural learning in humans, capuchins and chimpanzees [42]. In our study, the two-step task was transmitted horizontally and obliquely during the winter flocking period when juveniles were completely independent. It would be interesting to repeat the study during when fledglings forage with their parents under conditions of higher tolerance. Complex cultures are more often observed in species with extended parental care, such as orang-utans [80–82], and it has been argued that this is underpinned by the close tolerance and repeated opportunities for observation present between parent and offspring [83].

The maintenance of complex culture has long been hypothesized to rely on high fidelity, cognitively demanding social transmission mechanisms, such as imitation or teaching [30–32]. However, the results from this experiment point to a case in which a complex behaviour can emerge even in the absence of high fidelity copying, instead emerging through a combination of scaffolding and reconstruction within individuals. This calls into question the extent to which imitation contributes to the maintenance of human cultural behaviours, and speaks to the argument that a broader range of cognitive requirements can support complex culture [41,84]. Rather than faithful end-state copying [85], repeated interactions between individuals in a group, or individual trial-error learning, have been identified as alternative sources of stabilizing selection for modifications [86,87]. Further, a comparative transmission chain study of baboons and children using a non-copying task found that basic features of CCE arose from different cognitive mechanisms in humans and non-humans [40]. Thus, it may be that a variety of mechanisms can underlie the transmission of complex cultural behaviours, including the social learning of simpler behaviours and reconstruction of more complicated sets of behaviours (for a similar process in birdsong see [88], this issue). Anyone who has received professional training in a skill can attest to the critical role of individual learning through practice after initially observing a teacher.

Finally, in both the first and second generation of complex two-step learners, we observed increasing behavioural diversity at site and subpopulation level. This was beyond the diversity observed at the individual level, indicating that variation in preferences were aggregating as the number of individuals increased. However, diversity was still constrained well below what is expected by chance, suggesting an overall shared set of preferences. This is expected when the components of the two-step solution are socially learned, and thus constrain the subset of two-step variants available to be produced. The question inevitably arises whether this set of shared preferences constitute cultural traditions? While it would not be consistent with our original definition of a single, shared variant, such cultural polymorphisms are well accepted in other systems [89]. For example, passerine birds socially learn components of a song sequence, and while individuals may exhibit a unique subset of this sequence, vocabularies are still shared across populations as dialects [90]. If one accepts that diversity in behaviours arising from such a mechanism remains cultural in nature, one could also accept that the shared set of behaviours observed at the subpopulation level in great tits should constitute variants within a cultural lineage. Such questions require further research; for example, it would be interesting to investigate whether such diversity would be stable over time [89].

## 5. Conclusion

By applying diverse analytical approaches, including multi network-based diffusion analysis [65,70,91] and information-theoretic measures of cultural traits [78], we gained insight into: (i) how individuals reconstruct complex behaviours by recombining socially learned components, (ii) the importance of scaffolding to this process and (iii) how these constraints lead to a subset of shared preferences at the group level in the absence of imitation. Our study shows that while great tits can recombine previously socially learnt behaviours into more complex forms, they are unable to transmit complex traits in their entirety. Yet, this process of on-going social learning of components and asocial reconstruction still allowed complex foraging behaviours to be maintained in populations. Our findings highlight how simple mechanisms can lead to the emergence of complex behaviours in a small passerine species, even in the absence of learning mechanisms such as imitation.

## Data accessibility

All data and code for reproducing the analyses and figures in this manuscript are available on Github at https://github.com/michaelchimento/complex_from_simple (https://doi.org/10.5281/zenodo.5159069).

## References

[1] Whiten A. 2021 The burgeoning reach of animal culture. Science **372**, eabe6514. (10.1126/science.abe6514) 33795431

[2] Whiten A, Goodall J, McGrew WC, Nishida T, Reynolds V, Sugiyama Y, Tutin CE, Wrangham RW, Boesch C. 1999 Cultures in chimpanzees. Nature **399**, 682-685. (10.1038/21415) 10385119

[3] Thornton A, Samson J, Clutton-Brock T. 2010 Multi-generational persistence of traditions in neighbouring meerkat groups. Proc. R. Soc. B **277**, 3623-3629. (10.1098/rspb.2010.0611) PMC2982239 20610431

[4] Slater PJB. 1986 The cultural transmission of bird song. Trends Ecol. Evol. **1**, 94-97. (10.1016/0169-5347(86)90032-7) 21227788

[5] Aplin LM. 2019 Culture and cultural evolution in birds: a review of the evidence. Anim. Behav. **147**, 179-187. (10.1016/j.anbehav.2018.05.001)

[6] Whiten A, Ayala FJ, Feldman MW, Laland KN. 2017 The extension of biology through culture. Proc. Natl Acad. Sci. USA **114**, 7775-7781. (10.1073/pnas.1707630114) 28739924 PMC5544333

[7] Mesoudi A, Whiten A, Laland KN. 2006 Towards a unified science of cultural evolution. Behav. Brain Sci. **29**, 329-347; discussion 347-83. (10.1017/S0140525X06009083) 17094820

[8] Fehér O, Wang H, Saar S, Mitra PP, Tchernichovski O. 2009 De novo establishment of wild-type song culture in the zebra finch. Nature **459**, 564-568. (10.1038/nature07994) 19412161 PMC2693086

[9] Danchin E, Giraldeau L-A, Valone TJ, Wagner RH. 2004 Public information: from nosy neighbors to cultural evolution. Science **305**, 487-491. (10.1126/science.1098254) 15273386

[10] Parker KA, Anderson MJ, Jenkins PF, Brunton DH. 2012 The effects of translocation-induced isolation and fragmentation on the cultural evolution of bird song. Ecol. Lett. **15**, 778-785. (10.1111/j.1461-0248.2012.01797.x) 22590997

[11] Luther D, Baptista L. 2010 Urban noise and the cultural evolution of bird songs. Proc. R. Soc. B **277**, 469-473. (10.1098/rspb.2009.1571) PMC2842653 19846451

[12] Jesmer BR et al. 2018 Is ungulate migration culturally transmitted? Evidence of social learning from translocated animals. Science **361**, 1023-1025. (10.5061/dryad.8165qv5) 30190405

[13] Mueller T, O'Hara RB, Converse SJ, Urbanek RP, Fagan W. 2013 Social learning of migratory performance. Science **341**, 999-1002. (10.5061/dryad.1r0f7) 23990559

[14] Teitelbaum CS, Converse SJ, Fagan WF, Böhning-Gaese K, O'Hara RB, Lacy AE, Mueller T. 2016 Experience drives innovation of new migration patterns of whooping cranes in response to global change. Nat. Commun. **7**, 1-7. (10.1038/ncomms12793) PMC5025849 27597446

[15] Sasaki T, Biro D. 2017 Cumulative culture can emerge from collective intelligence in animal groups. Nat. Commun. **8**, 1-6. (10.1038/ncomms15049) 28416804 PMC5399285

[16] Berdahl AM, Kao AB, Flack A, Westley PAH, Codling EA, Couzin ID, Dell AI, Biro D. 2018 Collective animal navigation and migratory culture: from theoretical models to empirical evidence. Phil. Trans. R. Soc. B **373**, 20170009. (10.1098/rstb.2017.0009) 29581394 PMC5882979

[17] Henrich J, Boyd R, Derex M, Kline MA, Mesoudi A, Muthukrishna M, Powell AT, Shennan SJ, Thomas MG. 2016 Understanding cumulative cultural evolution. Proc. Natl Acad. Sci. USA **113**, E6724-E6725. (10.1073/pnas.1610005113) 27791123 PMC5098628

[18] Boyd R, Richerson PJ, Henrich J. 2011 Rapid cultural adaptation can facilitate the evolution of large-scale cooperation. Behav. Ecol. Sociobiol. **65**, 431-444. (10.1007/s00265-010-1100-3) 21423337 PMC3038225

[19] Mesoudi A, Thornton A. 2018 What is cumulative cultural evolution? Proc. R. Soc. B **285**, 20180712. (10.1098/rspb.2018.0712) PMC6015846 29899071

[20] Hobaiter C, Poisot T, Zuberbühler K, Hoppitt W, Gruber T. 2014 Social network analysis shows direct evidence for social transmission of tool use in wild chimpanzees. PLoS Biol. **12**, e1001960. (10.1371/journal.pbio.1001960) 25268798 PMC4181963

[21] Chimento M, Alarcón-Nieto G, Aplin LM. 2021 Population turnover facilitates cultural selection for efficiency in birds. Curr. Biol. **31**, 2477-2483. (10.1016/j.cub.2021.03.057) 33826905

[22] Gruber T, Chimento M, Aplin LM, Biro D. 2021 Efficiency fosters cumulative culture across species. Phil. Trans. R. Soc. B **377**, 20200308. (10.1098/rstb.2020.0308) 34894729 PMC8666915

[23] Whitehead H, Rendell L. 2014 The cultural lives of whales and dolphins. Chicago, IL: University of Chicago Press.

[24] Rendell L et al. 2010 Why copy others? Insights from the social learning strategies tournament. Science **328**, 208-213. (10.1126/science.1184719) 20378813 PMC2989663

[25] Taylor AH, Jelbert S. 2020 The crow in the room: New Caledonian crows offer insight into the necessary and sufficient conditions for cumulative cultural evolution. Behav. Brain Sci. **43**, e178. (10.1017/S0140525X20000102) 32772986

[26] Boyd R, Richerson PJ. 1996 Why culture is common, but cultural evolution is rare. Proc. Br. Acad. **88**, 77-93.

[27] Migliano AB et al. 2020 Hunter-gatherer multilevel sociality accelerates cumulative cultural evolution. Sci. Adv. **6**, eaax5913. (10.1126/sciadv.aax5913) 32158935 PMC7048420

[28] Cantor M, Chimento M, Smeele SQ, He P, Papageorgiou D, Aplin LM, Farine DR. 2021 Social network architecture and the tempo of cumulative cultural evolution. Proc. R. Soc. B **288**, 20203107. (10.1098/rspb.2020.3107) PMC7944107 33715438

[29] Derex M, Perreault C, Boyd R. 2018 Divide and conquer: intermediate levels of population fragmentation maximize cultural accumulation. Phil. Trans. R. Soc. B **373**, 20170062. (10.1098/rstb.2017.0062) 29440527 PMC5812974

[30] Tomasello M, Savage-Rumbaugh S, Kruger AC. 1993 Imitative learning of actions on objects by children, chimpanzees, and enculturated chimpanzees. Child Dev. **64**, 1688-1705. (10.2307/1131463) 8112113

[31] Tennie C, Call J, Tomasello M. 2009 Ratcheting up the ratchet: on the evolution of cumulative culture. Phil. Trans. R. Soc. B **364**, 2405-2415. (10.1098/rstb.2009.0052) 19620111 PMC2865079

[32] Lewis HM, Laland KN. 2012 Transmission fidelity is the key to the build-up of cumulative culture. Phil. Trans. R. Soc. B **367**, 2171-2180. (10.1098/rstb.2012.0119) 22734060 PMC3385684

[33] Legare CH. 2017 Cumulative cultural learning: development and diversity. Proc. Natl Acad. Sci. USA **114**, 7877-7883. (10.1073/pnas.1620743114) 28739945 PMC5544271

[34] Caldwell CA, Atkinson M, Blakey KH, Dunstone J, Kean D, Mackintosh G, Renner E, Wilks CEH. 2020 Experimental assessment of capacities for cumulative culture: review and evaluation of methods. Wiley Interdiscip. Rev. Cogn. Sci. **11**, e1516. (10.1002/wcs.1516) 31441239 PMC6916575

[35] Pargeter J, Khreisheh N, Stout D. 2019 Understanding stone tool-making skill acquisition: experimental methods and evolutionary implications. J. Hum. Evol. **133**, 146-166. (10.1016/j.jhevol.2019.05.010) 31358178

[36] Lucas AJ, Kings M, Whittle D, Davey E, Happé F, Caldwell CA, Thornton A. 2020 The value of teaching increases with tool complexity in cumulative cultural evolution. Proc. R. Soc. B **287**, 20201885. (10.1098/rspb.2020.1885) PMC7739508 33203332

[37] Whiten A, McGuigan N, Marshall-Pescini S, Hopper LM. 2009 Emulation, imitation, over-imitation and the scope of culture for child and chimpanzee. Phil. Trans. R. Soc. B **364**, 2417-2428. (10.1098/rstb.2009.0069) 19620112 PMC2865074

[38] Tennie C, Call J, Tomasello M. 2006 Push or pull: imitation vs. emulation in great apes and human children. Ethology **112**, 1159-1169. (10.1111/j.1439-0310.2006.01269.x)

[39] Tomasello M, Herrmann E. 2010 Ape and human cognition: what's the difference? Curr. Dir. Psychol. Sci. **19**, 3-8. (10.1177/0963721409359300)

[40] Saldana C, Fagot J, Kirby S, Smith K, Claidière N. 2019 High-fidelity copying is not necessarily the key to cumulative cultural evolution: a study in monkeys and children. Proc. R. Soc. B **286**, 20190729. (10.1098/rspb.2019.0729) PMC6571458 31161908

[41] Charbonneau M. 2020 Understanding cultural fidelity. Br. J. Phil. Sci. **71**, 1209-1233. (10.1093/bjps/axy052)

[42] Dean LG, Kendal RL, Schapiro SJ, Thierry B, Laland KN. 2012 Identification of the social and cognitive processes underlying human cumulative culture. Science **335**, 1114-1118. (10.1126/science.1213969) 22383851 PMC4676561

[43] Reindl E, Gwilliams AL, Dean LG, Kendal RL, Tennie C. 2020 Skills and motivations underlying children's cumulative cultural learning: case not closed. Palgrave Commun. **6**, 1-9. (10.1057/s41599-020-0483-7)

[44] Vale GL, Davis SJ, Lambeth SP, Schapiro SJ, Whiten A. 2017 Acquisition of a socially learned tool use sequence in chimpanzees: implications for cumulative culture. Evol. Hum. Behav. **38**, 635-644. (10.1016/j.evolhumbehav.2017.04.007) 29333058 PMC5765995

[45] Aplin L. 2016 Understanding the multiple factors governing social learning and the diffusion of innovations. Curr. Opin. Behav. Sci. **12**, 59-65. (10.1016/j.cobeha.2016.09.003)

[46] Estók P, Zsebok S, Siemers BM. 2010 Great tits search for, capture, kill and eat hibernating bats. Biol. Lett. **6**, 59-62. (10.1098/rsbl.2009.0611) 19740892 PMC2817260

[47] Morand-Ferron J, Cole EF, Rawles JEC, Quinn JL. 2011 Who are the innovators? A field experiment with 2 passerine species. Behav. Ecol. **22**, 1241-1248. (10.1093/beheco/arr120)

[48] Lefebvre L. 1995 The opening of milk bottles by birds: evidence for accelerating learning rates, but against the wave-of-advance model of cultural transmission. Behav. Process. **34**, 43-54. (10.1016/0376-6357(94)00051-H) 24897247

[49] Johannessen LE, Slagsvold T, Hansen BT. 2006 Effects of social rearing conditions on song structure and repertoire size: experimental evidence from the field. Anim. Behav. **72**, 83-95. (10.1016/j.anbehav.2005.09.019)

[50] Slagsvold T, Hansen BT, Johannessen LE, Lifjeld JT. 2002 Mate choice and imprinting in birds studied by cross-fostering in the wild. Proc. R. Soc. Lond. B **269**, 1449-1455. (10.1098/rspb.2002.2045) PMC1691058 12137574

[51] Slagsvold T, Wiebe KL. 2011 Social learning in birds and its role in shaping a foraging niche. Phil. Trans. R. Soc. B **366**, 969-977. (10.1098/rstb.2010.0343) 21357219 PMC3049099

[52] Slagsvold T, Wiebe KL. 2007 Learning the ecological niche. Proc. R. Soc. B **274**, 19-23. (10.1098/rspb.2006.3663) PMC1679873 17015332

[53] Aplin LM, Farine DR, Morand-Ferron J, Sheldon BC. 2012 Social networks predict patch discovery in a wild population of songbirds. Proc. R. Soc. B **279**, 4199-4205. (10.1098/rspb.2012.1591) PMC3441092 22915668

[54] Aplin LM, Farine DR, Morand-Ferron J, Cockburn A, Thornton A, Sheldon BC. 2015 Experimentally induced innovations lead to persistent culture via conformity in wild birds. Nature **518**, 538-541. (10.1038/nature13998) 25470065 PMC4344839

[55] Fragaszy D, Perry S. 2003 Towards a biology of traditions. In The biology of traditions (eds D Fragaszy, SE Perry), pp. 1-32. Cambridge, UK: Cambridge University Press.

[56] Perrins CM. 1979 British Tits. London, UK: Collins.

[57] Farine DR et al. 2015 The role of social and ecological processes in structuring animal populations: a case study from automated tracking of wild birds. R. Soc. Open Sci. **2**, 150057. (10.1098/rsos.150057) 26064644 PMC4448873

[58] Aplin LM, Sheldon BC, McElreath R. 2017 Conformity does not perpetuate suboptimal traditions in a wild population of songbirds. Proc. Natl Acad. Sci. USA **114**, 7830-7837. (10.1073/pnas.1621067114) 28739943 PMC5544276

[59] Whiten A, Mesoudi A. 2008 Establishing an experimental science of culture: animal social diffusion experiments. Phil. Trans. R. Soc. B **363**, 3477-3488. (10.1098/rstb.2008.0134) 18799418 PMC2607342

[60] Psorakis I, Roberts SJ, Rezek I, Sheldon BC. 2012 Inferring social network structure in ecological systems from spatio-temporal data streams. J. R. Soc. Interface **9**, 3055-3066. (10.1098/rsif.2012.0223) 22696481 PMC3479900

[61] Cairns SJ, Schwager SJ. 1987 A comparison of association indices. Anim. Behav. **35**, 1454-1469. (10.1016/S0003-3472(87)80018-0)

[62] Whitehead H, Dufault S. 1999 Techniques for analyzing vertebrate social structure using identified individuals: review and recommendations. Adv. Study Behav. **28**, 33-74. (10.1016/S0065-3454(08)60215-6)

[63] Hoppitt W, Farine D. 2018 Association indices for quantifying social relationships: how to deal with missing observations of individuals or groups. Anim. Behav. **136**, 227-238. (10.1016/j.anbehav.2017.08.029)

[64] Firth JA, Sheldon BC, Farine DR. 2016 Pathways of information transmission among wild songbirds follow experimentally imposed changes in social foraging structure. Biol. Lett. **12**, 20160144. (10.1098/rsbl.2016.0144) 27247439 PMC4938043

[65] Farine DR, Aplin LM, Sheldon BC, Hoppitt W. 2015 Interspecific social networks promote information transmission in wild songbirds. Proc. R. Soc. B **282**, 20142804. (10.1098/rspb.2014.2804) PMC4345451 25673683

[66] Farine DR. 2013 Animal social network inference and permutations for ecologists in R using *asnipe*. Methods Ecol. Evol. **4**, 1187-1194. (10.1111/2041-210X.12121)

[67] Butts CT. 2008 Social network analysis with sna. J. Stat. Software 24, 1-51. (10.18637/jss.v024.i06) PMC2447931 18618019

[68] Csárdi G, Nepusz T. 2006 The igraph software package for complex network research. InterJournal Complex Syst. **1695**, 1-9.

[69] Franz M, Nunn CL. 2009 Network-based diffusion analysis: a new method for detecting social learning. Proc. R. Soc. B **276**, 1829-1836. (10.1098/rspb.2008.1824) PMC2674490 19324789

[70] Hasenjager MJ, Leadbeater E, Hoppitt W. 2020 Detecting and quantifying social transmission using network-based diffusion analysis. J. Anim. Ecol. **90**, 8-26. (10.1111/1365-2656.13307) 32745269

[71] Hoppitt W, Boogert NJ, Laland KN. 2010 Detecting social transmission in networks. J. Theor. Biol. **263**, 544-555. (10.1016/j.jtbi.2010.01.004) 20064530

[72] Hoppitt WJE, Laland KN. 2013 Social learning: an introduction to mechanisms, methods, and models. Princeton, NJ: Princeton University Press.

[73] Hoppitt WJE, Photopoulou T, Hasenjager M, Leadbeater E. 2018 *NBDA: a package for implementing network-based diffusion analysis, 0.7.10 edition*. See http://github.com/whoppitt/NBDA.

[74] R Core Team. 2020 *R: A language and environment for statistical computing*. Vienna, Austria: R Foundation for Statistical Computing.

[75] Burnham K, Anderson D. 2002 Model selection and multi-model inference: a practical information-theoretic approach, 2nd edn. New York, NY: Springer.

[76] Morgan BJT. 2008 Applied stochastic modelling. New York, NY: Chapman and Hall/CRC.

[77] Lee W, Grimm KJ. 2018 Generalized linear mixed-effects modeling programs in R for binary outcomes. Struct. Equ. Model. **25**, 824-828. (10.1080/10705511.2018.1500141)

[78] Shannon CE. 1948 A mathematical theory of communication. Bell Syst. Techn. J. **27**, 379-423. (10.1002/j.1538-7305.1948.tb01338.x)

[79] Jacobs IF, Osvath M. 2015 The string-pulling paradigm in comparative psychology. J. Comp. Psychol. **129**, 89-120. (10.1037/a0038746) 25984937

[80] Jaeggi AV, Dunkel LP, van Noordwijk MA, Wich SA, Sura AAL, van Schaik CP. 2010 Social learning of diet and foraging skills by wild immature Bornean orangutans: implications for culture. Am. J. Primatol. **72**, 62-71. (10.1002/ajp.20752) 19790189

[81] Schuppli C, van Schaik CP. 2019 Animal cultures: how we've only seen the tip of the iceberg. Evol. Hum. Sci. **1**, 1-13. (10.1017/ehs.2019.1) PMC10427297 37588402

[82] Schuppli C, Graber SM, Isler K, van Schaik CP. 2016 Life history, cognition and the evolution of complex foraging niches. J. Hum. Evol. **92**, 91-100. (10.1016/j.jhevol.2015.11.007) 26989019

[83] van Schaik CP, Fragaszy D, Perry S. 2003 Local traditions in orangutans and chimpanzees: social learning and social tolerance. In The biology of traditions: models and evidence (eds RJ Fragaszy, DM Fragaszy, S Perry), pp. 297-328. Cambridge, UK: Cambridge University Press.

[84] Zwirner E, Thornton A. 2015 Cognitive requirements of cumulative culture: teaching is useful but not essential. Sci. Rep. **5**, 1-8. (10.1038/srep16781) PMC4660383 26606853

[85] Caldwell CA, Schillinger K, Evans CL, Hopper LM. 2012 End state copying by humans (*Homo sapiens*): implications for a comparative perspective on cumulative culture. J. Comp. Psychol. **126**, 161-169. (10.1037/a0026828) 22468937

[86] Biro D, Sasaki T, Portugal SJ. 2016 Bringing a time-depth perspective to collective animal behaviour. Trends Ecol. Evol. **31**, 550-562. (10.1016/j.tree.2016.03.018) 27105543

[87] Truskanov N, Prat Y. 2018 Cultural transmission in an ever-changing world: trial-and-error copying may be more robust than precise imitation. Phil. Trans. R. Soc. B **373**, 20170050. (10.1098/rstb.2017.0050) 29440516 PMC5812963

[88] Williams H, Lachlan R. 2021 Evidence for cumulative cultural evolution in bird song. Phil. Trans. R. Soc. B **377**, 20200322. (10.1098/rstb.2020.0322) 34894731 PMC8666912

[89] Tchernichovski O, Feher O, Fimiarz D, Conley D. 2017 How social learning adds up to a culture: from birdsong to human public opinion. J. Exp. Biol. **220**, 124-132. (10.1242/jeb.142786) 28057835 PMC5278621

[90] Tchernichovski O, Eisenberg-Edidin S, Jarvis ED. 2021 Balanced imitation sustains song culture in zebra finches. Nat. Commun. **12**, 1-4. (10.1038/s41467-021-22852-3) 33963187 PMC8105409

[91] Wild S, Allen SJ, Krützen M, King SL, Gerber L, Hoppitt WJE. 2019 Multi-network-based diffusion analysis reveals vertical cultural transmission of sponge tool use within dolphin matrilines. Biol. Lett. **15**, 20190227. (10.1098/rsbl.2019.0227) 31311483 PMC6685002

